# Socioeconomic, Behavioural, and Social Health Correlates of Optimism and Pessimism in Older Men and Women: A Cross-Sectional Study

**DOI:** 10.3390/ijerph20043259

**Published:** 2023-02-13

**Authors:** Heather Craig, Danijela Gasevic, Joanne Ryan, Alice Owen, John McNeil, Robyn Woods, Carlene Britt, Stephanie Ward, Rosanne Freak-Poli

**Affiliations:** 1 School of Public Health and Preventive Medicine, Monash University, Melbourne, VIC 3004, Australia; 2 Centre for Global Health, Usher Institute, University of Edinburgh, Teviot Place, Edinburgh EH8 9AG, UK; 3 Centre for Healthy Brain Ageing, School of Psychiatry, University of New South Wales, Sydney, NSW 2052, Australia; 4 Department of Geriatric Medicine, Prince of Wales Hospital, Randwick, NSW 2031, Australia; 5 School of Clinical Sciences at Monash Health, Monash University, Melbourne, VIC 3004, Australia

**Keywords:** optimism, pessimism, older adults, correlates, healthy ageing

## Abstract

Background: Optimism is a disposition characterised by positive future expectancies, while pessimism is characterised by expecting the worst. High optimism and low pessimism promote the health of older adults and may potentiate full engagement in life. We identified socioeconomic, behavioural, and social factors associated with optimism and pessimism in older adults. Methods: Participants included 10,146 community-dwelling, apparently healthy Australian adults aged 70 years and over from the ASPREE Longitudinal Study of Older Persons (ALSOP). Optimism and pessimism were measured using the revised Life Orientation Test. Cross-sectional ordinal logistic regression was used to determine the socioeconomic, behavioural, and social health factors associated with optimism and pessimism. Results: Higher education, greater physical activity, lower loneliness, and volunteering were associated with higher optimism and lower pessimism. Low social support was associated with higher pessimism. Higher socioeconomic advantage, greater income, and living alone were associated with lower pessimism. Women were more optimistic and less pessimistic than men. The association of age, smoking status, and alcohol consumption with optimism and pessimism differed for men and women. Conclusions: Factors associated with higher optimism and lower pessimism were also those demonstrated to support healthy ageing. Health-promotion action at the individual level (e.g., smoking cessation or regular physical activity), health professional level (e.g., social prescribing or improving access and quality of care for all older adults), and community level (e.g., opportunities for volunteer work or low-cost social activities for older adults) may improve optimism and reduce pessimism, possibly also promoting healthy ageing.

## 1. Introduction

The global population is ageing with the proportion of adults aged 65 years or older predicted to increase from 6% in 1990 to 16% by 2050 [1,2]. Healthy ageing entails more than simply being free of disease and disability [3]. To age in the healthiest way possible, a priority must be to enable older adults to continue to engage fully in life. Older adults have the right to live in and contribute to their community whatever their physical capacity [3]. Psychological and social wellbeing plays a significant role in healthy aging [4]. In alignment with this vision for health, a model of ‘positive epidemiology’ has recently been proposed [5]. As described by VanderWeele (2020): *“The study of diseases and risk factors should be supplemented with a “positive epidemiology” focused on health assets and a broader range of health-related states”* [5] (p. 189). Taking a ‘positive epidemiology’ approach will highlight opportunities to promote wellbeing, functional capacity, character strengths, and quality of life as our population ages by enhancing certain resources an individual possesses, including psychosocial characteristics [5]. Our work reflects the principles of health promotion that were first introduced by Aaron Antonovsky (1979) in his seminal work proposing ‘salutogenesis’—an asset-based approach to health and wellbeing focused on the factors that support wellness [6]. Optimism is a psychosocial factor that contributes to promoting health and wellbeing in older age [7].

Optimism is a facet of personality described as the tendency to expect favourable outcomes and to have a positive outlook [8]. Higher levels of optimism are associated with lower incidence of age-related diseases, such as cognitive impairment [9] and heart disease [10]. Additionally, optimism has been found to be positively correlated with quality of life [11]. Pessimism is described as expecting the worst [12] and historically has been considered diametrically opposed to optimism [13]. Higher levels of pessimism are associated with a greater risk of all-cause mortality [14], higher incidence of cardiovascular mortality [15], and with shorter leukocyte telomere length (a biomarker of cellular ageing) [16]. Evidence indicates that optimism and pessimism are modifiable [17]. It may be possible to identify modifiable factors associated with higher optimism and lower pessimism, which can be targeted in health-promotion interventions.

Studies report that younger age, more advantaged socioeconomic position, and higher level of education are each associated with higher optimism and lower pessimism [18,19,20]. Health-related behaviours may also play a role. For example, in young and middle-aged women, being physically active was associated with being more optimistic [21], while higher optimism is also associated with abstaining from smoking [22] and the probability of engaging in volunteer work [23], though there may be other factors that yet have not been sufficiently examined. There is some evidence on the putative association between lower pessimism and the increased likelihood of engaging in health-promoting behaviours [24], though less is known about whether healthy lifestyle behaviours in turn promote lower pessimism.

There is a scarcity of evidence for correlates of optimism and pessimism in older adults aged over 70 years. This age is of particular interest, as research suggests that levels of optimism and pessimism vary across the lifespan. Levels of optimism may decline at approximately 70 years, with a concurrent rise in levels of pessimism [18,25]. Identifying factors associated with low optimism or high pessimism when optimism and pessimism are assessed in later life could enable targeting interventions to help promote healthy ageing. Although recent evidence reports the association of lifestyle with optimism and longevity is not substantial [26], the unique association of different lifestyle factors and optimism and pessimism in both men and women is not clear. To address the above-mentioned gaps, we aim to determine the association of socioeconomic, behavioural, and social health factors with optimism and pessimism in men and women aged 70 years or over and to determine whether correlates of optimism and pessimism are distinct. Acknowledging that the current evidence is unclear regarding the endogenous or exogenous/environmental origin of personal optimism/pessimism, we interpret our findings as correlational connections rather than a cause–effect relationship of the socioeconomic, behavioural, and social health factors and optimism/pessimism on healthy ageing.

## 2. Materials and Methods

### 2.1. Study Population

The study population was men and women participating in the ASPREE Longitudinal Study of Older Persons (ALSOP). ALSOP was a sub-study of a large-scale clinical trial called ASPREE (Aspirin Reducing Events in the Elderly), which was designed to study the effects of a low dose of daily aspirin on health outcomes in older adults living in the United States of America (USA) and Australia. In early 2012, ASPREE participants who were living in Australia and had been recruited by general practitioners (primary care physicians) to participate in ASPREE were invited to participate in ALSOP [27,28]. The sample were aged 70 years and over, lived independently, and were free of cardiovascular disease, cognitive impairment, or major physical disability at baseline [26]. Findings of the ASPREE study have previously been published [29,30,31]. The ASPREE study complies with the Declaration of Helsinki (www.aspree.org) (accessed on 21 October 2022).

A total of 14,892 participants, 89% of ASPREE participants who were living in Australia participated in the ALSOP sub-study. ALSOP participants were representative of Australian adults who reached the age of 70 years in reasonably good health [28]. Of these, 12,896 individuals returned the baseline social questionnaire, which contained the revised Life Orientation Test (LOT-R), a test to measure optimism and pessimism [13]. The final sample used was those participants for whom we had data for all observations.

### 2.2. Assessment of Optimism and Pessimism

The LOT-R was used to assess optimism and pessimism. LOT-R is a validated tool consisting of six items plus four filler items (those not used to calculate optimism/pessimism scores), which were not included in the ALSOP study to reduce the burden on participants completing the survey, as per Kim et al. [32]. Of the six items, three items are positively worded, which are summed to assess dispositional optimism, whilst three items are negatively worded and summed to assess pessimism. Participants respond to questions on a 5-point Likert scale, from strongly disagree to strongly agree, with the optimism and pessimism subscale scores ranging from 3 to 15. Following recent evidence from Scheier et al. [33], our study considered optimism and pessimism as separate constructs. The LOT-R has been demonstrated to have good test–retest reliability and convergent and discriminant validity [13].

The optimism variable was not normally distributed, and none of the variable transformations (e.g., use of natural logarithm or log-10) corrected skewedness. Therefore, in line with previous literature [34,35], we expressed the optimism and pessimism variables as tertiles T1–T3, with higher tertiles representing greater optimism or pessimism.

### 2.3. Independent Variables

#### 2.3.1. Participant Characteristics

To summarize characteristics of the study population, age was considered in three categories, 70–74 years, 75–84 years, and 85 years and more. However, in the regression models, age was used as a continuous variable. Binary or categorical variables were created for gender (men or women), level of education (≤12 years—the formal school years or >12 years), marital status (married or divorced/widowed/never married), and living situation (with others or alone).

#### 2.3.2. Health-Related Behaviours

Binary categorical variables were created for physical activity (more—engaging in moderate or vigorous activity in a typical week or less—doing no, or only light, activity in a typical week), smoking status (assessed lifetime history of smoking tobacco as either never or current/former) and unpaid volunteer work (not including child-minding, babysitting, or caring; yes or no). Alcohol intake was categorized as never consumed alcohol, former drinker, current drinker—low risk; current drinker—high risk. Low risk was defined according to current Australian safe-drinking guidelines, which specify that safe alcohol consumption is ≤40 g pure ethanol (four standard drinks) on any one day and ≤100 g pure ethanol in a week [36].

#### 2.3.3. Socioeconomic Factors

Residential postcode determined the socio-economic indexes for areas (SEIFA) based on the Index of Relative Socio-economic Advantage and Disadvantage [37]. SEIFA was categorized in quintiles from least socio-economically advantaged to most advantaged. Annual household gross income was classified as <AUD 20,000; AUD 20,000–49,999; AUD 50,000–99,999; AUD 100,000+; or participants could also choose to respond to the income question with ‘prefer not to answer’.

#### 2.3.4. Social Health

Three measures of social health were included: social isolation, social support, and loneliness. Social isolation and social support were assessed using the ALSOP baseline social questionnaire, and loneliness was assessed at the ASPREE study baseline. Social isolation and social support were assessed using questions from the Revised Lubben Social Network Scale (LSNS), a validated scale of social health [38] (please see Additional Materials S1). To assess social isolation, participants were asked 3 questions: one question each on contact with friends and contact with relatives (they could choose from none, 1, 2, 3–4, 5–8, and 9 or more), and one question on frequency of attending community-based activities (a club, education class, or place of worship) (they could answer never, rarely—less than once per month, sometimes—one to three times per month, often—once a week or more, and always—most days). Social isolation was defined as attending community-based activities less than once a month (rarely), as well as having contact with four or fewer close friends and relatives per month. To assess social support, participants were asked four questions: one each about the number of friends and relatives with whom they could talk about private matters (they could choose from none, 1, 2, 3–4, 5–8, or 9 or more); and one each on the number of friends and relatives whom they could call upon for help (with the same options—i.e., none, 1, 2, 3–4, 5–8, or 9 or more). Social support was defined as having four or more close friends or relatives with whom participants could discuss private measures, as well as having friends or relatives who could be called upon for help. Those who answered at least one of the four questions on social support as “3–4 people” and the rest “none” were classified as socially supported. Loneliness was assessed via a single question of the Center for Epidemiological Studies—Depression (CES-D) scale [39]. Participants were asked to state how many days per week they felt lonely; they would be classified as lonely if they reported feeling lonely occasionally (3–5 days per week) or all of the time (5–7 days per week).

### 2.4. Statistical Analysis

Baseline characteristics of study participants were presented as counts and percentages for categorical variables and means (SD) for continuous variables. Based on prior evidence of gender differences in levels of optimism and pessimism in men and women [40,41], all analyses were stratified by gender. For simplicity, gender was coded as a binary variable: men (1) or women (2). As authors, we acknowledge the limitations of measuring gender as a binary variable; however, in the ASPREE study sex/gender were interchangeable. Participants nominated their sex (gender) as male or female.

Spearman correlations were used to explore the association between the independent variables (see Supplementary Material Table S1). The highest correlation between factors was 0.27 (for living situation and gender). It was, therefore, indicated that multicollinearity in the data is unlikely.

The association between the independent variables (socioeconomic, social health, and behavioural factors) and the dependent variables (optimism and pessimism) was tested using ordered logistic regression. While conceding the limitations of such a model, logistic regression enabled us to predict the likelihood of an individual being more/less optimistic (or pessimistic) based on the observed independent variables. The correlational study design means we indicate bidirectional relations, not causation (e.g., low optimism may influence loneliness, but similarly loneliness may influence low optimism). Calculating odds ratios allowed us to compare the impact of the various IVs on an individual being in a higher tertile of either optimism or pessimism rather than a lower tertile (that is, a category with a greater optimism or pessimism subscore—T3 or T2 compared to T1). The large sample size also influenced our choice of statistical model.

We also explored our results and compared our results with those of studies using a unidimensional measure of optimism (with the sum of items from the optimism subscale, and reverse-scored pessimism subscale items). We ran a separate ordered logistic regression to explore the association of the socioeconomic, social health, and behavioural factors with a unidimensional measure of optimism (Supplementary Material Table S3). Independent variables were mutually adjusted in the regression models. *p*-values of <0.05 were considered statistically significant. All statistical analyses were conducted using the software STATA Version 16.0 (StataCorp LLC., College Station, TX, USA).

### 2.5. Ethical Approval

The current project was reviewed and approved by Monash University Human Research Ethics committee, reference number: 21906. The ASPREE study complies with the Declaration of Helsinki and was approved by Monash University as the primary ethics site in Australia (www.aspree.org) (accessed 21 October 2022). Data used for analyses were based on version 3 of the longitudinal dataset. All participants provided informed written consent prior to taking part in the ASPREE and ALSOP studies.

## 3. Results

### 3.1. Baseline Participant Characteristics

Our study sample consisted of the 10,146 ALSOP participants for whom we had complete data for all observations (Supplementary Material Figure S1). The 2750 individuals for whom data were missing were more likely to be more pessimistic, older, female, not married, live alone, less socioeconomically advantaged, earn less than AUD 20,000 per year, less physically active, never smoked or drank alcohol, not do voluntary work, and not be lonely, when compared to those participants for whom data were complete (see Supplementary Material Table S2).

The average age of participants was 74.9 years (SD 4.13), with 52.0% women (Table 1). Women were more optimistic and less pessimistic than men. Men were more likely than women to have more than 12 years of education, be married, live with others, earn a higher annual income, be more physically active, a current or former smoker, drink alcohol at high risk levels, not do volunteer work, not be lonely, be socially isolated, and have low social support. Internal consistency of the LOT-R in our sample was calculated (Cronbach’s α: optimism subscale = 0.67; pessimism subscale = 0.80; unidimensional scale = 0.75).

### 3.2. Correlates of Optimism

#### 3.2.1. Factors Associated with Higher Optimism

For both men and women, having more than twelve years of education compared with twelve years or less (OR [95% CI]; men 1.13 [1.01–1.27]; women 1.26 [1.13–1.40]), being more physically active compared to less physically active (men 1.35 [1.19–1.52]; women 1.16 [1.05–1.29]), and doing volunteer work compared to not doing it (men 1.25 [1.12–1.39]; women 1.24 [1.12–1.38]) was associated with higher optimism.

#### 3.2.2. Factors Associated with Lower Optimism

**Figure 1:**
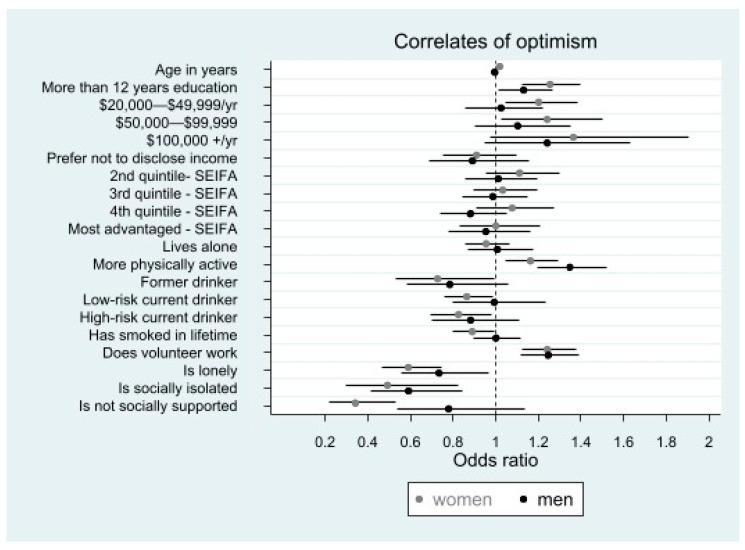
The association of socio-economic, behavioural, and social health factors with optimism in 10,146 men and women aged ≥70 years: the results of multivariable ordinal logistic regression. Footnote: Horizontal bars present 95%CIs. SEIFA: socio-economic indexes for areas Referent categories in order of variable appearance in the figure: <12 years education, <AUD 20,000/year, least advantaged, lives with others, less physically active, never drinks alcohol, never smoked, no volunteer work, not lonely, not socially isolated, and socially supported. As Figure 1 indicates, higher level of education, being more physically active, doing volunteer work, and not being lonely were associated with higher optimism compared to the referent categories for both men and women. Drinking alcohol at high risk levels was associated with lower optimism compared to the referent categories for men and women (Figure 1). For women, being older and earning AUD 20,000–99,999 compared with <AUD 20,000 was associated with higher optimism (Figure 1). Figure 1 also indicates that for women, being a current/former smoker, drinking alcohol at low-risk levels and having low social support were associated with lower optimism compared with the referent categories.

Being lonely compared with not being lonely (men 0.74 [0.56–0.97]; women 0.59 [0.47–0.74]) and being socially isolated compared with not being socially isolated (men 0.59 [0.42–0.84]; women 0.49 [0.29–0.83]) was associated with lower optimism (Figure 1, Supplementary Material Table S3).

#### 3.2.3. Gender Differences for Correlates of Optimism

Some associations with optimism were different for men and women. In women only, being older compared to being younger (1.02 [1.00–1.03]) or earning a higher annual household income (e.g., for AUD 50,000–99,999 compared to <AUD 20,000: 1.24 [1.03–1.50]) was associated with higher optimism. In women only, being a current or former smoker compared to those who never smoked (0.89 [0.80–1.00]), drinking alcohol at low-risk levels (0.87 [0.76–0.99]) or high-risk levels (0.83 [0.70–0.98]) compared to those who never drank alcohol or reporting low social support compared to being well socially supported (0.34 [0.22–0.53]) were associated with lower optimism.

### 3.3. Correlates of Pessimism

#### 3.3.1. Factors Associated with Higher Pessimism

For both men and women, being lonely compared to not being lonely (men 2.16 [1.52–3.06]), women 1.56 [1.23–1.97]) or having low social support compared to being well supported (men 1.73 [1.08–2.77], women 1.87 [1.23–2.86]) was associated with higher pessimism.

#### 3.3.2. Factors Associated with Lower Pessimism

**Figure 2:**
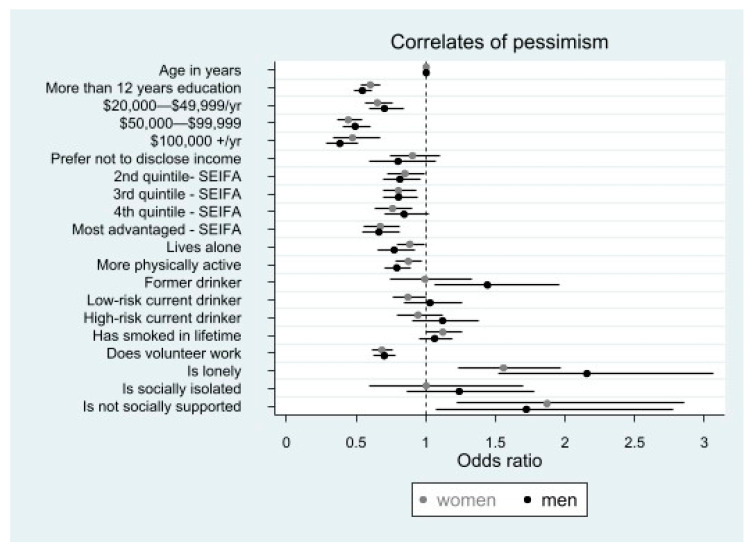
The association of socio-economic, behavioural, and social health factors with pessimism in 10,146 men and women aged ≥ 70 years: the results of multivariable ordinal logistic regression. Footnote: Horizontal bars present 95%CIs. SEIFA: socio-economic indexes for areas referent categories in order of variable appearance in the figure: <12 years education, <AUD 20,000/year, least advantaged, lives with others, less physically active, never drinks alcohol, never smoked, no volunteer work, not lonely, not socially isolated, and socially supported.

Having more than twelve years of education compared to having twelve years or less (men 0.55 [0.49–0.61]; women 0.60 [0.53–0.68]), being more physically active compared to less active(men 0.79 [0.70–0.89]; women 0.88 [0.79–0.98]), and doing volunteer work compared to not (men 0.70 [0.63–0.78]; women 0.69 [0.62–0.77]) was associated with lower pessimism (Figure 2). Living in a more socioeconomically advantaged neighbourhood compared to living in the least advantaged area (e.g., for the most advantaged area; men 0.66 [0.54–0.81]; women 0.67 [0.55–0.82]), earning a higher annual household income (e.g., for an income of more than AUD 100,000 men 0.38 [0.29–0.51]; women 0.48 [0.33–0.68]), and living alone compared to living with others (men 0.77 [0.65–0.92]; women 0.89 [0.80–0.99]) were associated with lower pessimism (Figure 2; Supplementary Material Table S3).

#### 3.3.3. Gender Differences for Correlates of Pessimism

Some correlates differed for men and women. In women only, living in the 4th quintile of neighbourhood socioeconomic advantage compared to the least advantaged area was associated with lower pessimism (0.76 [0.64–0.90]). For men only, being a former drinker compared to those who never drank alcohol was associated with higher pessimism (1.44 [1.07–1.96]).

As Figure 2 indicates, low social support and being lonely were associated with higher pessimism compared to the referent categories for both men and women. Higher level of education, being more physically active, doing volunteer work, living in a socioeconomically advantaged area, and earning AUD 20,000–49,999 were associated with lower pessimism compared to the referent categories for both men and women (Figure 2). For men, being a former alcohol drinker was associated with higher pessimism compared to the referent category (Figure 2). Figure 2 also indicates that for women only, living alone and drinking alcohol at low-risk amounts were associated with lower pessimism compared to the referent categories.

All the results were similar when a unidimensional measure of optimism was used (Supplementary Material Table S4).

## 4. Discussion

Our study aimed to identify the factors associated with optimism and pessimism in community dwelling older adults. While several factors were associated with both higher optimism and lower pessimism (higher education, more physical activity, less loneliness, and volunteering), several factors were associated with either higher optimism (less socially isolated) and lower pessimism (greater socioeconomic advantage, higher income, and living alone). This also supports our recommendation that optimism and pessimism are independent constructs. Women were more optimistic and less pessimistic than men, and more correlates were associated with optimism and pessimism among women. In women, higher optimism was associated with being older, having a higher household income, never smoking, not drinking alcohol, and having high social support, while lower pessimism was associated with living in a lower socioeconomic advantaged neighbourhood. For men, higher pessimism was associated with being a former alcohol drinker. Our conclusions remained similar when we employed a unidimensional measure of optimism (Supplementary Material Table S4).

### 4.1. Socioeconomic Correlates

Our result that greater annual income (for women) was associated with higher optimism aligns with previous research [19]. Similar to our study, in a study using a multifaceted definition of greater socioeconomic advantage encompassing area of residence and educational attainment, greater advantage was reportedly associated with lower pessimism for men and women [20].

We observed an association of higher level of education with higher optimism and lower pessimism. Evidence indicates that education level is associated with greater quality of life and improved living standards [42]. Individuals with higher level of formal education may have more positive expectations for the future because they are more likely to enjoy a better quality of life and higher living standards [42]. A higher level of education is also associated with greater participation in social activities [43] and lifelong learning [44]. This may contribute to increased optimism for those who are more educated. It would be advisable for governments to fund educational opportunities for older adults, such as the University of the Third Age in Victoria, Australia [45]. By providing accessible programs in the community to older adults, such as affordable group lessons, this may promote the prospects of this cohort increasing in their positive future outlook and the accompanying benefits to physical health.

In our study, earning a higher annual income and living in an area of greater socioeconomic advantage was associated with lower pessimism. This may be due to improved access to resources, which enhance wellbeing (housing, nutritious food, and adequate and comfortable clothing) [46]. Indeed, research suggests that adequate access to resources promotes optimism [47], thus further studies are needed to replicate this association with lower pessimism. Having sufficient access to material resources in the latter years of life may provide confidence to older adults. Those with adequate economic resources can enable older individuals to support themselves in the future when they are no longer working. Thus, they may have more positive future expectancies (i.e., higher optimism).

### 4.2. Behavioural Correlates

Our study reports that, in both genders, greater intensity of physical activity was associated with higher optimism and lower pessimism. Physical activity provides a sense of mastery, purpose, and self-esteem [21], which may influence optimism. Physical exercise also has mood-enhancing effects for older adults [48], boosting levels of serotonin, which also promotes good sleep [49]. Thus, the underlying mechanism explaining the association with greater physical activity and higher optimism and lower pessimism may be physiological as well as behavioural. We also report that drinking high-risk levels of alcohol was associated with lower optimism, though smoking cigarettes was not associated with optimism or pessimism. This could be because there are various social and environmental influences that influence why people begin to smoke, and the subsequent smoking is largely a manifestation of nicotine addiction [50]. The results related to smoking may need to be interpreted with caution as we do note that there was a relatively low number of current smokers in our cohort, which comprised individuals who had lived to 70 years or older free of chronic disabling disease.

We observed that healthy behaviours may promote higher optimism and lower pessimism. More optimistic individuals are also more likely to engage in health-promoting behaviours, including being physically active [51] and not smoking [22]. This is because optimistic people are more likely to believe that their actions will be successful—i.e., that their healthy lifestyle behaviours will result in improved wellbeing [52]. We acknowledge that reverse causality may contribute to our findings. In other words, the associations with healthy behaviours reported may reflect the effect of optimism and pessimism [53]. Our results contribute novel findings on the association of health-related behaviours with both optimism and pessimism in older adults.

### 4.3. Social Health Correlates

Social health describes “someone’s abilities to adapt in social situations and form satisfying meaningful relationships, and how someone interacts with and is supported by other people, institutions and services” [54]. The concepts of social isolation, loneliness, and social support are often discussed in relation to social health. Social isolation is an objective count of having few to no social relationships or social contact with others [55]. Social support is a subjective perception of the availability of resources from others, while loneliness is a subjective negative feeling of being isolated [55]. There is overwhelming evidence that poor social health is associated with a greater severity of chronic disease risk factors [56], lower quality of life [57,58], and mental ill health during cardiovascular disease recovery [59], as well as an increased risk of cardiovascular disease [60,61,62], dementia [62,63], and mortality [64]

We observed that older adults who do volunteer work or are less lonely are more likely to be more optimistic and less pessimistic, while low social support was associated with higher pessimism. Since social isolation, social support, and loneliness are considered independent predictors for depression, anxiety, cognitive decline, and even mortality in older adults [56,57,58,59,60,61,62,63,64], our novel results offer unique insights using a cluster of measurements of social health with future expectancies in older men and women.

Promoting optimism in older adults could be a promising intervention to improve social health as well as reduce the detrimental effects of poor social health. For example, the Best Possible Self intervention increases optimism [17], even among older adults [65]. Further, group activities addressing other associated factors could additionally be improving social interaction and thereby improving optimism/pessimism through social health. For example, affordable group educations, such as the University of the Third Age [45], could improve education and socialising—both identified factors in our study. Similarly, non-clinical group-based interventions prescribed through healthcare workers could target a range of other associated factors identified in our study. ‘Social prescribing’ can be defined as a process that enables healthcare professionals to refer patients to a link worker to initiate a non-clinical social prescription to improve their health and well-being [66,67]. Social prescribing is being proposed as a solution to poor social health, as an innovative holistic community-centred approach [68]. There are systematic reviews demonstrating benefits of social prescribing to well-being [69,70] and quality of life [71]. A recent systematic review identified that while there was a lack of evidence on social prescribing for chronic disease risk factors, the few group-exercise programs that were identified had benefits for physical activity [68]. The authors highlight that social prescribing could be effective in improving social health, as well as modifying the determinants of chronic diseases and promoting sustainable healthy behaviours. Here, we would recommend that optimism and pessimism be incorporated in such evaluations to provide a comprehensive evaluation of the potential benefits.

Volunteer work enables older individuals to keep busy, create meaning in life, and develop new social and role identities [72]. This may promote a positive outlook. Previous researchers have observed that people who are more optimistic and less pessimistic are more likely to do volunteer work [23], be less lonely [73], and have adequate social support [74]. We hypothesize that the relationship between the social health correlates measured and optimism and pessimism may be bidirectional.

### 4.4. Gender Differences

We observed that women reported higher optimism than men, which aligns with the findings of a study researching a German general population community sample aged 18 to 80 years [41]. In our study and others [18,40], women reported lower pessimism than men, while, in some studies, no gender difference in pessimism was observed ([18]—findings reported from the Dutch Longitudinal Internet Study for the Social Sciences (LISS) dataset; [41]). Our results contrast with studies reporting no difference in optimism for men and women, ([18]—findings reported from the Health and Retirement Study (HRS) dataset; [40]), and higher optimism in men compared to women ([18]—findings from the Dutch Longitudinal Internet Study for the Social Sciences (LISS) dataset). The divergence in results may reflect the underlying differences in personality that are seen in all men and women. Gender differences have been noted in temperament, emotions, and behaviour, such as aggression, as well as various indicators of psychological wellbeing [75]. Several major theories, including evolutionary theories and sociocultural theories, attempt to explain these gender differences [76]. Men and women’s personality may also be influenced by specific cultural aspects, such as gender roles, socialization, and gender equity, which could vary between study samples [76]. Gender differences in variability in optimism and pessimism through latter life are largely unknown, though in the Health and Retirement Study, the pessimism of men declined more slowly than women in the latter years of life [18]. Further research is needed to clarify potential gender variability in optimism with ageing.

Our study reports differences for the socioeconomic, behavioural, and social health correlates of both optimism and pessimism for men and women. For women, being older and reporting higher income was associated with higher optimism. For women, living in the 4th quintile of socioeconomic advantage was associated with lower pessimism. For men, being a former drinker was associated with higher pessimism. Our study also contributes unique results in that among women 70 years and older, those who were older were more likely to be optimistic than the younger women. Our results add novel insights into why women and men display different optimism and pessimism. It is plausible that in the populations sampled in previous studies, characteristic features, such as the age distribution, lifestyles, and socioeconomic position, of participants differed, thus explaining differences in the prevalence of higher optimism and lower pessimism of men and women. We report more socioeconomic, behavioural, and social health correlates of optimism and pessimism for women, compared to men. These differences may suggest that for women, life experience and socialization may play a more significant role in determining optimism and pessimism compared to men.

### 4.5. Implications

It has been demonstrated that optimism is associated with healthy ageing [7]; however, research into the biological mechanisms underlying this association is inconclusive [77,78]. Our study has identified several socioeconomic, behavioural, and social health factors that are associated with optimism and pessimism among relatively healthy older adults. However, with the current lack of evidence as to the exact origin of personal optimism/pessimism, interpretation is limited to the association and not causation. Even when analysing longitudinal data on optimism/pessimism, changes associated with measured optimism and/or pessimism may reflect other underlying traits or experiences that have been incurred outside the period of observation.

Nevertheless, our findings assist with progressing the literature in three facets. First, the associated factors can serve to identify high-risk groups, which could be targeted for improving optimism and pessimism. Second, interventions aiming to potentiate optimism and lessen pessimism could also serve to address these other factors if improvement in these factors also promotes optimism and decreases pessimism. Third, older adults (particularly women) eager to improve their optimism and pessimism could aim to address the modifiable associated factors, along with other strategies to promote wellbeing (e.g., mindfulness meditation).

Prior positive psychology interventions that also increase optimism have been shown to improve the mental and physical wellbeing of older adults and promote their independence [65,79]. However, at a population level, promoting healthy behaviours and positive social health for older adults may also increase optimism and decrease pessimism. This would enhance the many benefits associated with such lifestyles, as well as increase the odds of higher optimism and lower pessimism. For example, engaging in physical activity can also improve older adults’ global cognitive function [80] and build muscle-mass and strength [81]. Doing volunteer work can prevent premature mortality [82], as well as reduce depression and reduce loss of functional capacity [83]. More physically fit older adults are likely to be more optimistic and contribute to society through volunteering, which likely further promotes optimism. Campaigns to target one aspect of health have been shown to have modifying effects on others [84], which may also apply to factors related to ageing.

Further research will determine whether biological factors can explain possible gender differences between optimism and pessimism as people age. Our results suggest that health promoting measures, which target socioeconomic, behavioural, and social health correlates may possibly improve optimism/pessimism and perhaps the overall wellbeing of older adults. However, we nonetheless acknowledge the complexities and challenges in addressing adverse health issues faced in later life. Future research could tailor interventions for older adults to promote optimism and minimize pessimism so that adults might have increased chance of ageing in the healthiest way possible.

### 4.6. Strengths and Limitations

This is among the first studies of adults aged ≥70 years exploring the association of socioeconomic, behavioural, and social health variables with both optimism and pessimism in men and women. The cross-sectional study design means we were unable to determine causality between the independent covariates and optimism and pessimism. Therefore, our findings are to be interpreted as potential factors identified, which could improve optimism and pessimism among older adults; however, further research is required to ascertain this. Furthermore, our results may not be generalisable given that our study population have reached the age of ≥70 years free of life-limiting disease within 5 years, dementia, or major physical disability, and are living independently. Some adults face their later years with multimorbidity [85], and poor health is associated with lower optimism [25]. Selection bias may reflect the fact that most ALSOP participants were more optimistic, and optimistic people may be more willing to participate in a research study. Information gathered on potential correlates of optimism and pessimism was self-reported. Thus, reporting bias may be evident, particularly for behavioural measures (physical activity, alcohol intake, and smoking). We also acknowledge the potential consequences of performing multiple statistical tests, particularly increasing the type 1 error rate.

## 5. Conclusions

In community dwelling adults aged 70 years and over, those with favourable socioeconomic conditions, positive behavioural choices, and good social health are more likely to be more optimistic and less pessimistic. Older women are more likely to be optimistic and less likely to be pessimistic than older men, and more factors were associated with optimism and pessimism among women. The correlates identified can serve as a foundation to inform the development of interventions as we have identified high-risk groups which could be targeted, as well as opportunities to improve optimism and pessimism. Such interventions could not only promote optimism and reduce pessimism but also encourage health-promoting lifestyle behaviours and reduce social isolation and loneliness in adults aged 70 years and over.

## Figures and Tables

**Table 1:** Characteristics of 10,146 men and women aged 70 years and over.

	ALL: n = 10,146	Men: n = 4874	Women: n = 5272	*p* ^a^
Optimism subscore (Mean ± SD)	12.36 ± 2.34	12.25 ± 2.33	12.46 ± 2.34	<0.001
Pessimism subscore (Mean ± SD)	6.58 ± 3.10	6.85 ± 3.07	6.32 ± 3.10	<0.001
Unidimensional optimism score (Mean ± SD)	23.78 ± 4.47	23.39 ± 4.40	24.13 ± 4.50	<0.001
Age (Mean ± SD)	74.9 ± 4.13	74.9 ± 4.17	74.9 ± 4.10	0.65
Age group (years) n (%)				
70–74	6260 (61.7)	3034 (62.2)	3226 (61.2)	0.43
75–84	3590 (35.4)	1694 (34.8)	1896 (36.0)	
85+	296 (2.92)	146 (3.0)	150 (2.8)	
Education level n (%)				
≤12 years	5768 (56.9)	2618 (53.7)	3150 (59.7)	<0.001
>12 years	4378 (43.2)	2256 (46.3)	2122 (40.3)	
Marital status n (%)				
Not married	3633 (35.8)	1078 (22.1)	2555 (48.5)	<0.001
Married	6513 (64.2)	3796 (77.9)	2717 (51.5)	
Living situation n (%)				
Lives with others	7218 (71.1)	4080 (83.7)	3138 (59.5)	<0.001
Lives alone	2928 (28.9)	794 (16.3)	2134 (40.5)	
SEIFA ^1^ n (%)				
Least advantaged	2119 (20.9)	1009 (20.7)	1110 (21.1)	0.48
2nd quintile	2057 (20.3)	994 (20.4)	1063 (20.2)	
3rd quintile	2938 (29.0)	1381 (28.3)	1557 (29.5)	
4th quintile	1719 (16.9)	855 (17.5)	864 (16.4)	
Most advantaged	1313 (12.9)	635 (13.0)	678 (12.9)	
Annual gross household income (AUD) n (%)				
<AUD 20,000	1465 (14.4)	558 (11.4)	907 (17.2)	<0.001
AUD 20,000–49,999	5326 (52.5)	2543 (52.2)	2783 (52.8)	
AUD 50,000–99,999	1895 (18.7)	1130 (23.2)	765 (14.5)	
AUD 100,000+	468 (4.6)	315 (6.5)	153 (2.9)	
Prefer not to answer	992 (9.8)	328 (6.7)	664 (12.6)	
Physical activity n (%)				
Less physically active ^2^	3386 (33.4)	1280 (26.3)	2106 (39.9)	<0.001
More physically active ^3^	6760 (66.6)	3594 (73.7)	3166 (60.1)	
Smoking status n (%)				
Never	5589 (55.1)	2105 (43.2)	3484 (66.1)	<0.001
Current/former	4557 (44.9)	2769 (56.8)	1788 (33.9)	
Alcohol intake n (%)				
Never drank alcohol	1455 (14.3)	398 (8.2)	1057 (20.0)	<0.001
Former drinker	461 (4.5)	254 (5.2)	207 (3.9)	
Current—low risk ^4^	5571 (54.9)	2495 (51.2)	3076 (58.3)	
Current—high risk ^5^	2659 (26.2)	1727 (35.4)	932 (17.7)	
Volunteer work n (%)				
No	5770 (56.9)	2892 (59.3)	2878 (54.6)	<0.001
Yes	4376 (43.1)	1982 (40.7)	2394 (45.4)	
Loneliness n (%)				
Not lonely	9670 (95.3)	4670 (95.8)	5000 (94.8)	0.02
Lonely	476 (4.7)	204 (4.2)	272 (5.2)	
Social isolation n (%)				
Not socially isolated	9949 (98.1)	4746 (97.4)	5203 (98.7)	<0.001
Socially isolated	197 (1.9)	128 (2.6)	69 (1.4)	
Social support n (%)				
Socially supported	9950 (98.1)	4764 (97.7)	5186 (98.4)	0.02
Low social support	196 (1.9)	110 (2.3)	86 (1.6)	

^a^*p*-value for difference between men and women. ^1^: SEIFA: the socio-economic indexes for areas based on the Index of Relative Socio-economic Advantage and Disadvantage (ABS, 2016); ^2^: less physically active—doing no, or only light, activity in a typical week; ^3^: more physically active—engaging in moderate or vigorous activity in a typical week; ^4^: low risk—≤40 g pure ethanol (four standard drinks) on any one day and ≤100 g pure ethanol in a week; ^5^—>40 g pure ethanol on any one day or >100 g pure ethanol in a week.

## Data Availability

The dataset supporting the conclusions are not publicly available for legal and ethical reasons. Data cannot be shared publicly as data are part of a large ongoing observational cohort with a rigorous process to access data. Data are available from Monash University for researchers who meet the criteria (contact via https://aspree.org/aus/for-researchers; aspree@monash.edu) (accessed 21 October 2022).

## Supplementary Materials

The following supporting information can be downloaded at: https://www.mdpi.com/article/10.3390/ijerph20043259/s1.
