# Supplementary material for: Socioeconomic, Behavioural, and Social Health Correlates of Optimism and Pessimism in Older Men and Women: A Cross-Sectional Study
Source: Int J Environ Res Public Health. 2023 Feb 13;20(4):3259. doi: 10.3390/ijerph20043259 (PMC9961087; doi:10.3390/ijerph20043259)
Supplement: Supplementary file 1 [file ijerph-20-03259-s001.zip › ijerph-2193817-supplementary.pdf]

Socioeconomic, behavioural and social health correlates of optimism and pessimism in older men and women: a cross-sectional study
Additional material

File name: "2023_Optimism_Correlates_SUPPMATERIAL"

Format: MS Word (docx)

Socioeconomic, behavioural and social health correlates of optimism and pessimism in older men and women: a cross-sectional study

Supplementary Material

Contains four (4) tables to detail further analyses completed to support the conclusions presented in this manuscript, plus one figure detailing selection of final study sample included in analyses, and additional material on the specific items used to measure the optimism and pessimism, and social health variables (i.e., loneliness, social isolation, and social support)

Table S1. Spearman correlations between independent variables, n=10,146

	**1**	**2**	**3**	**4**	**5**	**6**	**7**	**8**	**9**	**10**	**11**	**12**
**1**	1.00											
**2**	0.01	1.00										
**3**	-0.05	-0.06	1.00									
**4**	0.18	0.27	-0.04	1.00								
**5**	-0.03	-0.01	0.25	-0.04	1.00							
**6**	-0.10	-0.07	0.18	-0.19	0.22	1.00						
**7**	-0.12	-0.15	0.06	-0.07	0.05	0.08	1.00					
**8**	-0.03	-0.22	0.08	-0.07	0.09	0.09	0.07	1.00				
**9**	-0.03	-0.22	0.08	-0.07	0.02	0.06	0.06	-0.02	1.00			
**10**	0.04	0.02	-0.03	0.19	-0.02	-0.05	-0.05	-0.01	-0.02	1.00		
**11**	-0.001	-0.05	-0.03	0.02	-0.04	-0.03	-0.05	-0.01	-0.07	0.03	1.00	
**12**	0.004	-0.02	-0.02	0.03	-0.01	-0.03	-0.05	-0.002	-0.05	0.03	0.26	1.00

Key: 1 = age (years); 2= gender; 3 = education level; 4 = living situation; 5 = SEIFA - the Socio-Economic Indexes for areas based on the Index of Relative Socio-economic Advantage and Disadvantage (ABS, 2016); 6 = Annual gross household income ($AU); 7 = physical activity; 8 = alcohol intake; 9 = volunteer work; 10 = loneliness; 11 = social isolation; 12 = social support

Table S2. Characteristics of study participants with and without complete data on variables of interest

	Complete data available (n = 10,146):	Missing data on variables of interest (n = 2,750):	p
**Optimism (Mean ± SD)**	12.36 ± 2.34	12.42 ± 2.33	0.26
**Pessimism (Mean ± SD)**	6.58 ± 3.10	7.14 ± 3.23	<0.001
**Age (Mean ± SD)**	74.9 ± 4.13	76.5 ± 4.77	<0.001
**Age group (years) n(%):**			<0.001
**70 – 74**	6260 (61.7)	1290 (46.7)	
**75 – 84**	3590 (35.4)	1290 (46.7)	
**85 +**	296 (2.9)	170 (6.2)	
**Gender n(%):**			
**men**	4874 (48.0)	1010 (36.7)	<0.001
**women**	5272 (52.0)	1740 (63.3)	
**Education Level n(%):**			
**≤ 12 years**	5768 (56.8)	1853 (67.4)	<0.001
**> 12 years**	4378 (43.2)	897 (32.6)	
**Married n(%):**			
**no**	3633 (35.8)	1219 (44.3)	<0.001
**yes**	6513 (64.2)	1531 (55.7)	
**Living situation n(%):**			
**lives with others**	7218 (71.1)	1835 (66.7)	<0.001
**lives alone**	2928 (28.9)	915 (33.3)	
**SEIFA[1] n(%):**			
**least advantaged**	2119 (20.9)	695 (25.6)	<0.001
**2nd quintile**	2057 (20.3)	567 (20.9)	
**3rd quintile**	2938 (29.0)	714 (26.3)	
**4th quintile**	1719 (16.9)	445 (16.4)	
**most advantaged**	1313 (12.9)	297 (10.9)	

**Annual gross household income ($AU) n(%):**			
**< $20,000**	1465 (14.4)	419 (20.4)	<0.001
**$20,000 – 49,999**	5326 (52.5)	1004 (48.9)	
**$50,000 – 99,999**	1895 (18.7)	262 (12.8)	
**$100,000 +**	468 (4.6)	70 (3.4)	
**prefer not to answer**	992 (9.8)	298 (14.5)	
**Physical activity n(%):**			
**less physically active[2]**	3386 (33.4)	912 (35.9)	0.02
**more physically active[3]**	6760 (66.6)	1630 (64.1)	
**Smoking status n(%):**			
**never**	5589 (55.1)	1633 (59.4)	<0.001
**current/former**	4557 (48.9)	1117 (40.6)	
**Alcohol intake n(%):**			
**never drank alcohol**	1455 (14.3)	557 (20.3)	<0.001
**former drinker**	461 (4.5)	136 (4.9)	
**current – low risk[4]**	5571 (54.9)	1432 (52.1)	
**current – high risk[5]**	2659 (26.2)	625 (22.7)	
**Volunteer work n(%):**			
**no**	5770 (56.9)	1524 (61.3)	<0.001
**yes**	4376 (43.1)	961 (38.7)	
**Lonely n(%):**			
**no**	9670 (95.3)	2582 (94.0)	0.005
**yes**	476 (4.7)	165 (6.0)	
**Socially isolated n(%):**			
**not socially isolated**	9949 (98.1)	1533 (98.0)	0.91
**socially isolated**	197 (1.9)	31 (2.0)	
**Social support n(%):**			
**supported**	9950 (98.1)	2385 (97.8)	0.44
**low**	196 (1.9)	53 (2.2)	

[1]: SEIFA: the socio-economic indexes for areas based on the Index of Relative Socio-economic Advantage and Disadvantage (ABS, 2016); [2] :less physically active– doing no, or only light, activity in a typical week; [3]: more physically active– engaging

in moderate or vigorous activity in a typical week; [4]: low risk - ≤40g pure ethanol (four standard drinks) on any one day, and ≤100g pure ethanol in a week; [5] - > 40g pure ethanol on any one day or > 100g pure ethanol in a week

Total n (missing):

SEIFA = 2718; Income = 2054; Physical activity = 2542; Volunteer work = 2485; Lonely = 2747; Social isolation = 1564; Social support = 24

Table S3. The association of socioeconomic, behavioural and social health factors with optimism and pessimism in 10,146 men and women aged 70 years and over: results of ordinal logistic regression

	**Optimism**		**Pessimism**	
	**Men**<br>**OR (95% CI)**<br>**p-value**	**Women**<br>**OR (95% CI)**<br>**p-value**	**Men**<br>**OR (95% CI)**<br>**p-value**	**Women**<br>**OR (95% CI)**<br>**p-value**
**Age**	1.00 (.98 – 1.01)	**1.02 (1.00 – 1.03)**	1.00 (0.99 – 1.02)	1.01 (0.99 – 1.02)
	0.64	**0.008**	0.47	0.45
**Education level**				
**≤ 12 years**	1.00	1.00	1.00	1.00
**> 12 years**	**1.13 (1.01 - 1.27)**	**1.26 (1.13 – 1.40)**	**0.55 (0.49 – 0.61)**	**0.60 (0.53 – 0.68)**
	**0.03**	**<0.001**	**<0.001**	**<0.001**
**SEIFA[1]:**				
**least advantaged**	1.00	1.00	1.00	1.00
**2nd quintile**	1.01 (0.86 – 1.19) 0.91	1.12 (0.95 – 1.31)	**0.82 (0.69 – 0.96)**	**0.85 (0.72 – 1.00)**
		0.17	**0.02**	**0.04**
**3rd quintile**	0.99 (0.84 – 1.15)	1.03 (0.90 – 1.20)	**0.88 (0.69 – 0.94)**	**0.81 (0.70 – 0.93)**
	0.84	0.64	**0.007**	**0.004**
**4th quintile**	0.88 (0.74 – 1.05)	1.08 (0.91 – 1.28)	0.85 (0.70 – 1.02)	**0.76 (0.64 – 0.90)**

	0.15	0.38	0.09	**0.002**
**most advantaged**	0.95 (0.78-1.16)	1.00 (0.83 – 1.21)	**0.66 (0.54 – 0.81)**	**0.67 (0.55 – 0.82)**
	0.63	0.98	**<0.001**	**<0.001**
**Annual gross household income ($AU) :**				
**< $20,000**	1.00	1.00	1.00	1.00
**$20,000 – 49,999**	1.02 (0.86 – 1.22)	**1.20 (1.04 – 1.38)**	**0.70 (0.59 – 0.84)**	**0.65 (0.56 – 0.76)**
	0.79	**0.01**	**<0.001**	**<0.001**
**$50,000 – 99,999**	1.10 (0.90 – 1.35)	**1.24 (1.03 – 1.50)**	**0.49 (0.40 – 0.61)**	**0.44 (0.36 – 0.54)**
	0.35	**0.03**	**<0.001**	**<0.001**
**$100,000 +**	1.24 (0.95 – 1.63)	1.36 (0.97 – 1.90)	**0.38 (0.29 – 0.51)**	**0.48 (0.33 – 0.68)**
	0.12	0.07	**<0.001**	**<0.001**
**prefer not to answer**	0.89 (0.69 – 1.16)	0.91 (0.75 – 1.10)	0.80 (0.60 – 1.07)	0.91 (0.75 – 1.10)
	0.39	0.33	0.13	0.32
**Living situation:**				
**lives with others**	1.00	1.00	1.00	1.00
**lives alone**	1.01 (0.87- 1.17)	0.96 (0.86 – 1.07)	**0.77 (0.65 – 0.92)**	**0.89 (0.89 – 0.99)**
	0.90	0.41	**0.004**	**0.04**
**Physical activity:**				
**less physically active[2]**	1.00	1.00	1.00	1.00
**more physically active[3]**	**1.35 (1.19 – 1.52)**	**1.16 (1.05 – 1.29)**	**0.79 (0.70 – 0.89)**	**0.88 (0.79 – 0.98)**

	**<0.001**	**0.005**	**<0.001**	**0.02**
**Smoking status:**				
**never**	1.00	1.00	1.00	1.00
**current/former**	1.00 (0.90 – 1.12)	**0.89 (0.80 – 1.00)**	1.07 (0.95 – 1.19)	1.12 (1.00 – 1.26)
	0.96	**0.04**	0.26	0.05
**Alcohol intake:**				
**never**	1.00	1.00	1.00	1.00
**former drinker**	0.78 (0.58 – 1.06)	0.87 (0.65 – 1.16)	**1.44 (1.07 – 1.96)**	1.00 (0.75 – 1.33)
	0.11	0.35	**0.02**	.98
**current – low risk[4]**	0.99 (0.80 – 1.24)	**0.87 (0.76 – 0.99)**	1.03 (0.84 – 1.26)	0.87 (0.76 – 1.00)
	0.95	**0.03**	0.78	0.05
**current – high risk[5]**	0.88 (0.70 – 1.11)	**0.83 (0.70 – 0.98)**	1.12 (0.90 – 1.38)	0.95 (0.79 – 1.13)
	0.28	**0.03**	0.30	0.54
**Volunteer work:**				
**no**	1.00	1.00	1.00	1.00
**yes**	**1.25 (1.12 – 1.39)**	**1.24 (1.12 – 1.38)**	**0.70 (0.63 – 0.78)**	**0.69 (0.62 – 0.77)**
	**<0.001**	**<0.001**	**<0.001**	**<0.001**
**Lonely:**				
**no**	1.00	1.00	1.00	1.00
**yes**	**0.74 (0.56 – 0.97)**	**0.59 (0.47 – 0.74)**	**2.16 (1.52 – 3.06)**	**1.56 (1.23 – 1.97)**

	**0.03**	**<0.001**	**<0.001**	**<0.001**
**Socially isolated:**				
**not socially isolated**	1.00	1.00	1.00	1.00
**socially isolated**	**0.59 (0.42 – 0.84)**	**0.56 (0.34 – 0.94)**	1.24 (0.86 – 1.78)	1.00 (0.59 – 1.70)
	**0.004**	**0.03**	0.25	0.99
**Social support:**				
**socially supported**	1.00	1.00	1.00	1.00
**low**	0.78 (0.54 – 1.14)	**0.33 (0.21 – 0.52)**	**1.73 (1.08 – 2.77)**	**1.87 (1.23 – 2.86)**
	0.20	**<0.001**	**0.02**	**0.004**

[1] SEIFA: the socio-economic indexes for areas based on the Index of Relative Socio-economic Advantage and Disadvantage (ABS, 2016); [2] less physically active– doing no, or only light, activity in a typical week; [3] more physically active– engaging in moderate or vigorous activity in a typical week; [4] low risk - ≤40g pure ethanol (four standard drinks) on any one day, and ≤100g pure ethanol in a week; [5] high risk - > 40g pure ethanol on any one day or > 100g pure ethanol in a week

* Interpretation: T1 vs. T2 + T3, or T1 + T2 vs. T3, that is the odds of being in a higher category of optimism or pessimism

Table S4. Socioeconomic, behavioural and social health correlates of optimism and pessimism, using a unidimensional measure of optimism (n=10,146)

	**Optimism***	
	**Men**	**Women**
	**OR (95% CI)**	**OR (95% CI)**
	**p**	**p**
**Age**	1.00 (0.99-1.01)	1.00 (0.99-1.02)
	0.80	0.73
**Education level:**		
**≤ 12 years**	1.00	1.00
**> 12 years**	**1.64 (1.46-1.84)**	**1.58 (1.42-1.76)**
	**<0.001**	**<0.001**
**SEIFA[1]:**		
**least advantaged**	1.00	1.00
**2nd quintile**	1.08 (0.92-1.28)	**1.18 (1.01-1.38)**
	0.34	**0.04**
**3rd quintile**	1.13 (0.97-1.32)	1.22 (1.05-1.41)
	0.12	0.008
**4th quintile**	1.08 (0.90-1.28)	**1.31 (1.11-1.55)**
	0.41	**0.002**
**most advantaged**	**1.25 (1.02-1.52)**	**1.32 (1.09-1.59)**
	**0.03**	**0.004**
**Annual gross household income ($AU):**		
**< $20,000**	1.00	1.00
**$20,000-49,999**	**1.37 (1.15-1.64)**	**1.52 (1.32-1.75)**
	**<0.001**	**<0.001**

**$50,000-99,999**	**1.78 (1.45-2.18)**	**1.99 (1.65-2.41)**
	**<0.001**	**<0.001**
**$100,000+**	**1.99 (1.51-2.62)**	**2.13 (1.51-2.99)**
	**<0.001**	**<0.001**
**prefer not to answer**	1.04 (0.79-1.35)	1.00 (0.82-1.20)
	0.80	0.97
**Living situation:**		
**lives with others**	1.00	1.00
**lives alone**	1.14 (0.98-1.33)	1.03 (0.93-1.15)
	0.10	0.55
**Physical activity:**		
**less physically active[2]**	1.00	1.00
**more physically active[3]**	**1.33 (1.18-1.50)**	**1.18 (1.07-1.31)**
	**<0.001**	**0.002**
**Smoking status:**		
**never**	1.00	1.00
**current/former**	0.94 (0.84-1.05)	**0.89 (0.79-0.99)**
	0.24	**0.04**
**Alcohol intake:**		
**never**	1.00	1.00
**former drinker**	**0.73 (0.54-0.99)**	0.96 (0.72-1.27)
	**0.04**	0.78
**current – low risk[4]**	0.99 (0.81-1.21)	1.02 (0.89-1.17)
	0.95	0.79
**current – high risk[5]**	0.88 (0.71-1.08)	0.93 (0.78-1.11)
	0.23	0.41
**Voluntary work:**		

**no**	1.00	1.00
**yes**	**1.43 (1.29-1.60)**	**1.43 (1.29-1.59)**
	**<0.001**	**<0.001**
**Lonely:**		
**no**	1.00	1.00
**yes**	**0.56 (0.42-0.75)**	**0.60 (0.47-0.76)**
	**<0.001**	**<0.001**
**Socially isolated:**		
**not socially isolated**	1.00	1.00
**socially isolated**	**0.63 (0.43-0.92)**	0.90 (0.55-1.47)
	**0.02**	0.68
**Social support:**		
**socially supported**	1.00	1.00
**low**	**0.43 (0.29-0.64)**	**0.38 (0.24-0.59)**
	**<0.001**	**<0.001**

[1]: SEIFA: the socio-economic indexes for areas based on the Index of Relative Socio-economic Advantage and Disadvantage (ABS, 2016); [2] less physically active– doing no, or only light, activity in a typical week; [3] more physically active– engaging in moderate or vigorous activity in a typical week; [4] low risk - ≤40g pure ethanol (four standard drinks) on any one day, and ≤100g pure ethanol in a week; [5] high risk - > 40g pure ethanol on any one day or > 100g pure ethanol in a week

* Interpretation: T1 vs. T2 + T3, or T1 + T2 vs. T3, that is the odds of being in a higher category of overall ‘optimism’ score (when 3 optimism items are summed with the reverse-scored 3 pessimism item

Figure S1. Process of selection of final sample for data analysis

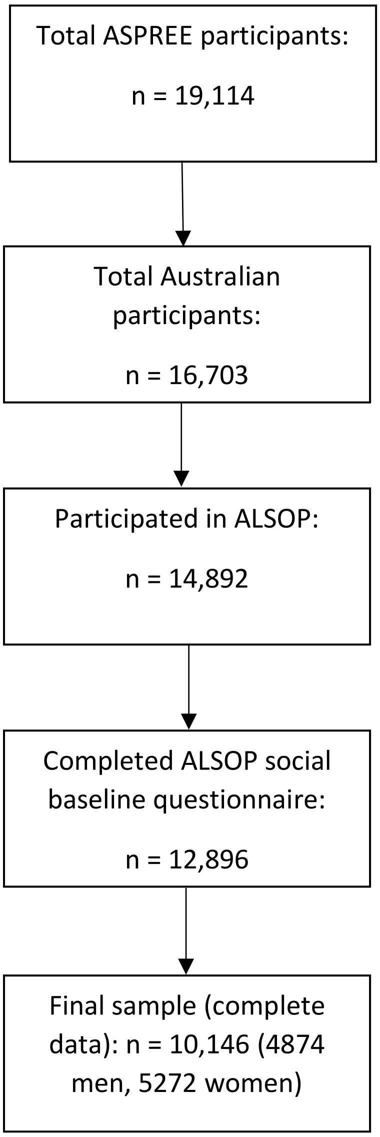

ASPREE: ASPirin in Reducing Events in the Elderly clinical trial

ALSOP: ASPREE Longitudinal Study of Older Persons study

Additional Material S1. Specific items used to measure the optimism and pessimism, and social health variables (i.e., loneliness, social isolation, and social support)

a. Items from Revised Life Orientation Test (LOT-R) (Scheier et al., 1994) included in ALSOP Social Baseline questionnaire

Optimism items
1. In uncertain times, I usually expect the best
2. I'm always optimistic about my future
3. Overall, I expect more good things to happen to me than bad
**Pessimism items**
1. If something can go wrong for me, it will
2. I hardly ever expect things to go my way
3. I rarely count on good things happening to me

note: to highlight the optimism and pessimism items, they do not appear in the order in which they were presented in the ALSOP social baseline questionnaire

b. Questions to assess social isolation and social support from Lubben Social Network Scale (J.E. Lubben, 1988), included in the ALSOP Social Baseline questionnaire

**FAMILY: considering the people to whom you are related either by birth of marriage**					
1. How many relatives (including spouses, partners, children, etc.) do you see or hear from at least once a month?					
none	1	2	3-4	5-8	9 or more
2. How many relatives do you feel at ease with, that you can talk to about private matters?					
none	1	2	3-4	5-8	9 or more
3. How many relatives do you feel close to, such that you could call on them for help?					
none	1	2	3-4	5-8	9 or more
**FRIENDS: considering ALL your friends, including those who live in your neighbourhood**					
4. How many friends do you see or hear from at least once a month?					
none	1	2	3-4	5-8	9 or more

5. How many friends do you feel at ease with, that you can talk to about private matters?					
none	1	2	3-4	5-8	9 or more
6. How many friends do you feel close to, such that you could call on them for help?					
none	1	2	3-4	5-8	9 or more

c. Question assessing loneliness, from CES-D (L. S. Radloff, 1977), included in the ASPREE baseline data

**I felt lonely**			
0<br>Rarely or <1/day	1<br>Some or a little, 1-2 days	2<br>Occasionally or moderate, 3-4 days	3<br>All of the time, 5-7 days
